# The regulatory network of epithelial-mesenchymal transition-associated non-coding RNAs in thyroid cancer: molecular mechanisms, clinical implications, and therapeutic strategies

**DOI:** 10.3389/fonc.2025.1592467

**Published:** 2025-06-02

**Authors:** Rui Zhao, Jiaju Tian, Jing Peng, Ruiting Ma, Songbo Fu

**Affiliations:** ^1^ The First Clinical Medical College, Lanzhou University, Lanzhou, China; ^2^ Department of Endocrinology, First Hospital of Lanzhou University, Lanzhou, China; ^3^ Gansu Provincial Endocrine Disease Clinical Medicine Research Center, Lanzhou, China

**Keywords:** thyroid cancer, epithelial-mesenchymal transition, non-coding RNAs, miRNAs, circRNAs, lncRNAs, diagnostic biomarkers

## Abstract

Thyroid cancer represents the most prevalent malignant neoplasm within the endocrine system, exhibiting a steadily increasing global incidence. Non-coding RNAs (ncRNAs) have emerged as a pivotal focus in thyroid cancer research, demonstrating significant involvement in tumor progression through epigenetic regulation. Contemporary studies reveal widespread dysregulation of ncRNA expression profiles in thyroid malignancies, where differentially expressed ncRNAs exert either tumor-suppressive or oncogenic functions by modulating epithelial-mesenchymal transition (EMT) mechanisms. This review synthesizes current knowledge on EMT-related ncRNA mechanisms driving thyroid carcinogenesis and evaluates their diagnostic and therapeutic potential. By elucidating these molecular interactions, we aim to catalyze discovery of novel ncRNA-mediated pathways, advance targeted treatment strategies, and ultimately enhance clinical outcomes for thyroid cancer patients.

## 1 Introduction

Thyroid cancer, a malignancy arising from follicular epithelial or parafollicular C-cells of the thyroid gland, constitutes the most prevalent endocrine malignancy in head and neck oncology. Recent epidemiological data from the United States (2024) document 44,020 new thyroid cancer diagnoses (2.2% of total cancer incidence) with 2,170 mortality events (0.355% of cancer-related deaths) (1). Histopathological classification delineates three principal subtypes: Differentiated Thyroid Carcinoma [DTC; encompassing papillary (PTC), follicular (FTC), and Hürthle cell variants], Medullary Thyroid Carcinoma (MTC), and Anaplastic Thyroid Carcinoma (ATC). DTC predominates, representing 84% of thyroid malignancies (2, 3). While most DTC cases exhibit favorable prognoses, clinical challenges persist for patients with advanced differentiation grades and those presenting with poorly differentiated/undifferentiated histotypes, who demonstrate elevated risks of recurrence and metastasis (4). Emerging evidence identifies multifactorial etiology involving ionizing radiation exposure, dietary iodine levels, metabolic disorders (obesity/diabetes), environmental factors (food additives), and tobacco use alongside diagnostic surveillance biases (5, 6).

Epithelial-Mesenchymal Transition (EMT), an evolutionarily conserved biological process, serves as a critical driver of tumor cell invasiveness and metastatic dissemination, substantially complicating therapeutic interventions and prognostic predictions. This multistep phenotypic plasticity is orchestrated through synergistic molecular crosstalk among multiple signaling pathways (7), enabling epithelial cells to adopt mesenchymal characteristics. Characteristic transformations include loss of apical-basal polarity, dissolution of adherens junctions, and augmented migratory capacity. While physiologically essential during embryogenesis, EMT becomes pathologically activated in disease states, particularly facilitating cancer progression through enhanced tissue invasion and distant metastasis (8).

Concurrently, non-coding RNAs (ncRNAs) have emerged as a focus of intense investigation in oncological research, with particular relevance to thyroid cancer pathogenesis and therapeutic innovation. Defined as functionally active RNA species lacking protein-coding potential, ncRNAs exert regulatory control over gene expression networks, intracellular signaling cascades, and oncogenic transformation (9). Mechanistic studies demonstrate that EMT induction in thyroid malignancies is critically modulated by specific ncRNA signatures, establishing direct links between ncRNA dysregulation and metastatic progression (10). Consequently, EMT-associated ncRNAs present dual diagnostic and therapeutic potential. Elucidating the precise molecular circuitry through which ncRNAs govern EMT dynamics promises to reveal novel targets for metastasis suppression and recurrence prevention. Such advancements hold translational significance for optimizing clinical management strategies, potentially elevating both survival outcomes and quality-of-life metrics in thyroid cancer patients.

## 2 EMT

EMT describes a biological process wherein epithelial cells progressively acquire mesenchymal characteristics through coordinated loss of apical-basal polarity and intercellular adhesion properties (11). Originally conceptualized by Greenberg and Hay in 1982 (12), this fundamental mechanism has subsequently been shown to mediate both physiological processes (e.g., embryonic morphogenesis and wound healing) and pathological tumor dissemination. EMT activation triggers dissolution of epithelial tight junction complexes coupled with cytoskeletal reorganization and upregulation of motility-associated proteins (13). These molecular alterations induce characteristic morphological changes from cuboidal to spindle-shaped cells with pseudopodia formation and front-rear polarity establishment, enabling chemotaxis-driven tumor cell migration that facilitates metastatic spread (14).

### 2.1 Characteristics of the EMT process

#### 2.1.1 Progressivity

Studies demonstrate that EMT progresses through multiple intermediate states, displaying gradations of epithelial and mesenchymal markers between polarized epithelial and fully mesenchymal phenotypes (11, 13, 15). Single-cell analyses reveal that hybrid epithelial/mesenchymal states predominate in human tissues, whereas complete mesenchymal conversion rarely occurs in both physiological and neoplastic contexts (16). Notably, epithelial-derived malignancies acquire migratory competence through tumor-associated dedifferentiation mechanisms, directly facilitating metastatic dissemination (17). The partial EMT state—characterized by incomplete mesenchymal transformation—confers three critical oncogenic properties: (i) enhanced motility, (ii) bidirectional phenotypic plasticity, and (iii) lineage reprogramming capacity (18). Intratumoral heterogeneity analyses further suggest that epithelial-dominant subclones exhibit elevated malignant potential and preferential metastatic tropism compared to mesenchymal counterparts (19, 20).

#### 2.1.2 Reversibility

Epithelial-mesenchymal transition demonstrates bidirectional plasticity, wherein epithelial and mesenchymal phenotypes undergo reciprocal conversion mediated by microenvironmental cues (13). Notably, clinical evidence documents that circulating tumor cells in distant metastases frequently retain epithelial markers (11), contradicting the classical EMT paradigm predicting complete mesenchymal transformation. This apparent paradox may be reconciled through mesenchymal-epithelial transition (MET) mechanisms, whereby metastasized mesenchymal-like cells regain epithelial characteristics to facilitate metastatic niche formation. While EMT activation is essential for initiating metastatic dissemination, its maintenance proves dispensable during subsequent colonization phases.

### 2.2 Mechanisms

The regulatory network governing epithelial-mesenchymal transition involves a dynamic, hierarchical system driven by coordinated integration of extracellular microenvironmental cues, intracellular signaling pathway activation, transcription factor cascades, and epigenetic modifications across multiple biological scales (21–23).

#### 2.2.1 External stimuli and tumor microenvironment

The precise temporal dynamics underlying metastatic initiation in primary tumors remain incompletely understood. Notably, EMT activation is primarily mediated through TME-derived stress signaling, with key drivers encompassing hypoxia, TGF-β pathway activation, pro-inflammatory cytokines (e.g., IL-6), growth factors (e.g., EGF), and extracellular matrix remodeling (24). These factors coordinately promote tumor cell dissociation from primary sites and subsequent migration via interlinked molecular cascades (25).

##### 2.2.1.1 Hypoxia/hypoxia-inducible factor-1α

Hypoxia-Inducible Factor-1α (HIF-1α) is one of the key microenvironmental factors that induce cancer metastasis. Under hypoxic conditions, HIF-1α is up-regulated and regulates the expression of EMT markers and a variety of EMT transcription factors (26), including Snail, Twist1, ZEB1, ZEB2, and SIP1. HIF-1α up-regulation is significantly associated with lymph node metastasis and distant metastasis, and is involved in tumor growth, metabolism, angiogenesis, and metastasis by regulating gene expression (27). At the chromatin level, HIF-1α regulates the expression of EMT markers through histone modifications. For example, H3K4 acetylation (H3K4Ac) marks the promoters of EMT-related genes or transcription factors and promotes the repression of epithelial genes and activation of mesenchymal genes, such as E-cadherin and poikilodulin. It has been demonstrated that HIF-1α overexpression induces EMT, down-regulates the epithelial marker E-cadherin and up-regulates the mesenchymal marker waveform proteins, and correlates with a highly aggressive and metastatic phenotype (28). In addition, IL-11 promotes hypoxia-induced EMT through HIF-1α induction and enhances the invasive and migratory capacity of undifferentiated thyroid cancer (ATC) cells (29). In the thyroid cancer cell line FTC133, HIF-1α overexpression increased Twist gene expression, whereas inhibition of HIF-1α activity abrogated the increase in Twist gene expression under hypoxic conditions (30). In addition, hypoxia mediates immunosuppression, and HIF-1α inhibits the cGAS-STING pathway through activation of anti-apoptotic genes, up-regulation of PD-L1, activation of the CD39/CD73 pathway, and up-regulation of miR25/93, thereby conferring the ability of tumor cells to evade immune surveillance (24).

##### 2.2.1.2 TGF-β family proteins: inducers of EMT

Transforming growth factor-β1 (TGF-β1) serves as a master regulator of EMT, orchestrating tumor invasion and metastasis through three molecular tiers (1): transcriptional reprogramming, (2) post-transcriptional modulation, and (3) translational control (31, 32).

Within Smad-dependent signaling, ligand-bound TGF-β receptors phosphorylate Smad2/3 and Smad1/5/8, which subsequently form heteromeric complexes with Smad4 that undergo nuclear translocation. These nuclear complexes coordinate with EMT master regulators (Snail1/2, ZEB1/2, Twist) to suppress epithelial markers (e.g., E-cadherin) and drive cellular plasticity. Notably, Smad3 specifically governs the Snail1/2-ZEB1/2-Twist transcriptional axis through promoter binding affinity modulation, establishing an auto-amplification circuit for sustained EMT activation.

Non-canonical pathways engage PI3K-Akt-mTOR and MAPK cascades, executing chromatin topology reorganization, epigenetic landscape reshaping, alternative mRNA splicing, and miRNA-mediated gene silencing to fine-tune EMT progression (24, 33, 34). Exogenous TGF-β triggers exosome-mediated intercellular communication, significantly suppressing papillary thyroid carcinoma (TPC1) proliferation via EMT- and stemness-related marker induction. Mechanistically, platelet-derived TGF-β coordinates Smad/NF-κB crosstalk to potentiate EMT-driven metastatic dissemination.

#### 2.2.2 Core signaling networks

##### 2.2.2.1 Wnt/β-catenin pathway

The Wnt protein family comprises evolutionarily conserved, multidomain glycoproteins encoded by polycistronic genes, forming intricate receptor-mediated signaling networks critical for embryonic morphogenesis and tissue homeostasis (35). Central to stem cell maintenance and regenerative processes, the Wnt/β-catenin pathway operates through spatiotemporally coordinated ligand-receptor interactions.

Nineteen identified Wnt ligands exhibit spatiotemporal-specific expression patterns mediated by autocrine/paracrine mechanisms (36). Their biogenesis initiates with endoplasmic reticulum (ER)-lumenal palmitoylation catalyzed by membrane-bound O-acyltransferase PORCN, enabling hydrophobic modification essential for secretory trafficking. Mature Wnt proteins complex with the cargo receptor Wntless (WLS), subsequently mediating intercellular communication via vesicular transport systems and regulated exocytosis.

The Wnt signaling network bifurcates into two principal transduction cascades: (1) Canonical Pathway (β-catenin-dependent): Ligand-receptor engagement inhibits β-catenin degradation complexes, enabling cytoplasmic β-catenin stabilization and subsequent nuclear import. Nuclear β-catenin forms transcriptional complexes with T-cell factor/lymphoid enhancer factor (TCF/LEF) to activate metastasis-associated gene programs (37); (2) Non-canonical Pathway (β-catenin-independent): Frizzled (FZD) co-receptors with LRP5/6 activate Dishevelled phosphoprotein clusters, orchestrating planar cell polarity (PCP) signaling and mechanotransduction-regulated migratory phenotypes.

##### 2.2.2.2 PI3K/AKT/mTOR pathway

Phosphatidylinositol 3-kinase (PI3K), a lipid-modifying enzyme in the phosphoinositide 3-kinase family, orchestrates oncogenic progression through dual regulation of mitogenic signaling and metabolic reprogramming. Receptor tyrosine kinase (RTK)-initiated extracellular cues undergo RAS-mediated activation, facilitating membrane recruitment of class IA PI3K catalytic subunit p110α. This enzymatic subunit converts phosphatidylinositol-4,5-bisphosphate (PIP2) to phosphatidylinositol-3,4,5-trisphosphate (PIP3), thereby initiating AKT/mTOR signaling cascades critical for tumor survival.

The mammalian target of rapamycin (mTOR), a serine/threonine kinase, functions as a metabolic gatekeeper by integrating growth factor inputs, nutrient availability, and bioenergetic states. Mechanistically, mTOR complex 2 (mTORC2) phosphorylates AKT at Ser473 and protein kinase C (PKC) isoforms, thereby coordinating cytoskeletal reorganization essential for EMT-driven cell motility (38). TGF-β receptors engage PI3K regulatory subunit p85 to activate AKT signaling, which subsequently triggers mTORC1-dependent translational control mechanisms that potentiate EMT progression (39, 40).

#### 2.2.3 Transcription factor network

The EMT is coordinately regulated by core transcription factors, primarily comprising the SNAIL, TWIST, and zinc-finger E-box binding (ZEB) families (41). These transcriptional regulators are activated through canonical signaling pathways including Wnt and TGF-β, subsequently initiating signaling cascades that drive EMT progression.

Key transcriptional regulators such as TWIST1/2, inhibitor of differentiation (ID) proteins, and E12/E47 can induce EMT through individual or synergistic mechanisms. TWIST1, a basic helix-loop-helix (bHLH) transcription factor, mediates both embryonic morphogenesis and pathological EMT by dual regulation of cadherin switching: suppressing E-cadherin while promoting N-cadherin expression (42).

The SNAIL zinc-finger family (SNAI1/SLUG) exerts transcriptional repression of E-cadherin through specific binding to E-box motifs in its promoter. During embryogenesis, SNAIL signaling facilitates epithelial cell delamination and subsequent mesenchymal differentiation. Clinically relevant, this pathway’s dysregulation correlates with metastatic progression in malignancies such as papillary thyroid carcinoma and melanoma. Experimental models demonstrate that in BRAF-mutant thyroid carcinomas undergoing dedifferentiation, SNAI1/ZEB1/ZEB2 overexpression induces: (1) marked suppression of tight junction/desmosome-related genes; and (2) concurrent upregulation of intermediate filament and basement membrane components - a molecular signature validated across multiple studies (43–45).

ZEB1 is a pleiotropic transcription factor belonging to a family of zinc finger and homology frame transcription factors located on chromosome 10p11.2.ZEB1 coordinates gene transcription through the RAS/ERK, TGF-β, PI3K/Akt, and NF-κB signaling pathways, regulates key developmental processes and drives tumorigenesis and metastasis.

#### 2.2.4 Epigenetic and post-transcriptional regulation

Epigenetic modifications dynamically modulate access to EMT-associated genes via multiple pathways, including DNA methylation (e.g., hypermethylation of the E-cadherin promoter) and histone acetylation/deacetylation (e.g., H3K4Ac). Many studies have focused on investigating heritable molecular factors independent of DNA sequence changes, including DNA methylation, histone modifications, noncoding RNAs, and chromatin remodeling (46). DNA methylation markers linked to colorectal cancer (CRC) (e.g., SFRP1, SFRP2, SDC2) can regulate transcriptional elements and non-coding RNA production, playing a key role in EMT (47). For example, deacetylated TWIST1 (K73/76) interacts with the NuRD deacetylase complex, recruiting it to repress epithelial gene transcription, while acetylated TWIST1 (acK73/76) interacts with BRD8 and the TIP60 complex to activate MYC transcription (48). In metastatic cancers, GRHL1 expression is downregulated, which may reduce epithelial cell adhesion and promote a migratory phenotype (49). The epigenetic state of EMT intermediates is synergistically maintained across multiple regulatory levels, exhibiting the typical components and limitations of complex networks (50). For instance, the miR-200 family inhibits EMT by targeting ZEB1/2, and long noncoding RNAs (e.g., MUF) enhance EMT via Wnt/β-catenin or TGF-β signaling. Additionally, epigenetic feedback in ZEB1 loops and random biomolecule allocation during cell division contribute to solid tumor cells’ EMT resistance (51).

#### 2.2.5 Cellular phenotype remodeling

The activation of EMT induces profound morphological alterations and molecular reprogramming in cancer cells. This process is characterized by upregulated expression of mesenchymal markers including N-cadherin, vimentin, fibroblast-specific protein 1 (FSP1), and fibronectin, concomitant with downregulation of epithelial markers such as E-cadherin and occludin (11, 14, 52). E-cadherin, a transmembrane protein localized at intercellular adherens junctions and basolateral membranes, serves as a critical regulator for maintaining epithelial integrity. Its downregulation disrupts adherens junction assembly, while reduced occludin expression compromises tight junction integrity, ultimately resulting in cell junction complex disassembly. Notably, N-cadherin exhibits weaker homotypic binding affinity compared to E-cadherin, conferring enhanced cellular motility and invasive properties. Cytoskeletal reorganization manifests through diminished cytokeratin expression and elevated vimentin levels, which coordinately regulate intracellular trafficking and membrane protein dynamics (14). The dissolution of apical-basal polarity enables establishment of front-rear polarity, facilitating integrin-mediated matrix engagement during invasive progression. This polarity transition promotes pseudopodia formation and directional migration through Rho GTPase-dependent mechanisms. Collectively, these coordinated molecular and architectural adaptations substantially augment cancer cell metastatic competence.

## 3 EMT and TC

Thyroid surgery, radioactive iodine therapy, and thyroid-stimulating hormone (TSH) suppression remain the primary treatment modalities for thyroid cancer (53). Research indicates that TC displays significant heterogeneity in EMT-related regulatory factors and cell signaling pathways. This heterogeneity is characterized by altered cell morphology, decreased cell adhesion, and enhanced migratory capacity (54). These alterations facilitate tumor invasion and metastasis. The metastatic cascade involves three key steps: invasion of surrounding tissues by tumor cells, intravasation of tumor cells into the circulatory system through endothelial cells, and colonization of distal tissues with subsequent formation of metastatic foci.

### 3.1 EMT and the regulation of invasiveness in TC

**Figure 1:**
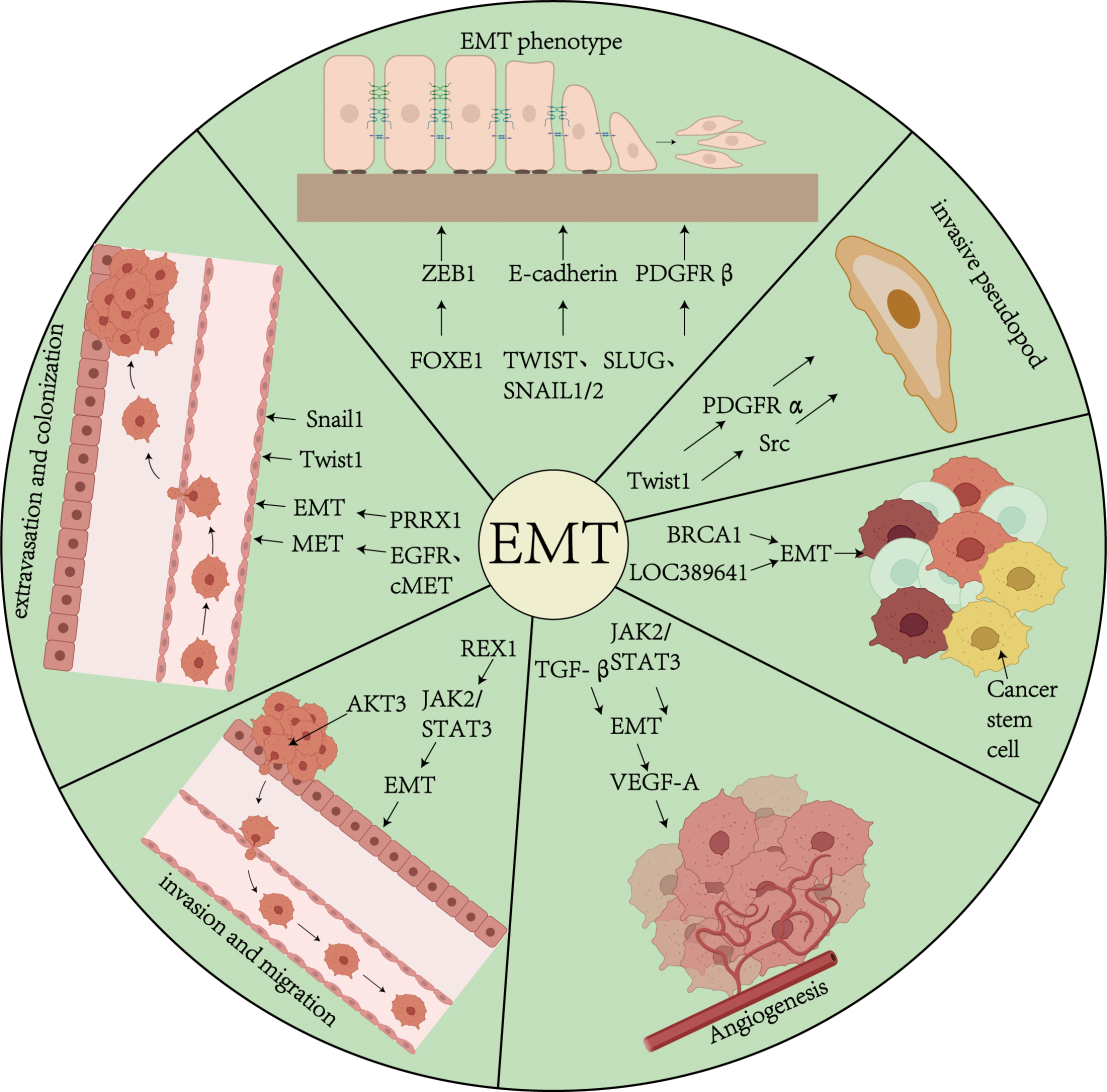
Overview of EMT and Tumors. This figure demonstrates the role of biological processes of EMT in tumor progression in thyroid cancer. EMT affects tumor progression through a variety of pathways and mechanisms, including the TGF-β signaling pathway, transcription factors (Snail, Twist, ZEB1/2), etc., which drive the transformation of epithelial cells to a mesenchymal phenotype, and promote the formation of cellular pseudopods, proliferation of tumor stem cells, angiogenesis, invasion and colonization.

Tumor invasion represents a pathological progression wherein malignant cells breach endothelial barriers through active migration or passive dissemination, infiltrating adjacent normal tissues and establishing distant metastases. This process is defined by cellular polarity loss, architectural disorganization, and acquisition of migratory/invasive capabilities (55). Significantly, within primary tumor microenvironments, the epithelial-mesenchymal transition program is preferentially activated in discrete neoplastic subpopulations. Histopathological evaluations reveal pronounced EMT phenotypes at tumor-stroma interfaces adjacent to desmoplastic connective tissue. These regions exhibit characteristic molecular signatures including downregulation of E-cadherin coupled with aberrant activation of transcriptional repressors (Snail, Slug, ZEB1, ZEB2) and the Twist1 basic helix-loop-helix transcription factor. Mechanistically, Twist1 drives invasive pseudopod formation through coordinated induction of platelet-derived growth factor receptor α (PDGFRα) expression and Src kinase activation, thereby potentiating tumor cell invasion (11) (**Figure 1**).

Emerging insights into thyroid carcinogenesis highlight pivotal EMT regulatory networks. FOXE1 has been demonstrated to directly bind the ZEB1 promoter, enhancing its transcriptional activation to drive EMT-mediated migratory and invasive phenotypes (56). This FOXE1-ZEB1 signaling axis constitutes a critical regulatory pathway in thyroid cancer EMT. Conversely, BRCA1 exerts tumor-suppressive effects via dual mechanisms: (1) upregulating E-cadherin to preserve epithelial integrity, and (2) concurrently suppressing mesenchymal markers (vimentin, PDGFRβ, p-PKCα, TWIST, ZEB1), effectively inhibiting dedifferentiation and reducing invasive potential (57).

Recent investigations further elucidate microRNA-mediated EMT modulation. miR-200c post-transcriptionally targets the 3’ untranslated region (UTR) of parathyroid hormone-like hormone (PTHLH) mRNA, attenuating proliferation, invasion, and EMT progression in anaplastic thyroid carcinoma (ATC) models (58).

### 3.2 EMT and migration of TC

Tumor cell migration is the process by which cancer cells disseminate from the primary tumor to other organs via the vascular or lymphatic systems (55). Cancer stem cells (CSCs) and metastatic cells possess the ability to promote angiogenesis, which supports their tumorigenicity and metastatic potential. EMT-mediated angiogenesis is considered a mechanism that facilitates the entry of tumor cells into the systemic circulation. The upregulation of vascular endothelial growth factor A (VEGF-A), a protein that promotes neoangiogenesis, enhances tumor angiogenesis, thereby providing additional nutrients and oxygen to support tumor growth. EMT not only activates genes associated with cell differentiation, motility, and adhesion but also induces the expression of genes involved in angiogenesis regulation, including VEGF-A. Additionally, ZEB1, through its zinc finger domain, directly binds to E-box elements, thereby initiating the EMT process, enhancing cancer cell stemness and migration, and promoting chemoresistance (59) (**Figure 1**).

Studies have shown that TGF-β transiently upregulates VEGF-A expression (60). In thyroid cancer, the JAK2/STAT3 signaling pathway not only directly induces the EMT process and enhances cancer cell migration but also synergistically promotes VEGF-A secretion, thereby regulating tumor metastasis and angiogenesis. Dong quai methylin downregulates the JAK2/STAT3 signaling pathway, thereby inhibiting VEGF-A expression to limit angiogenesis (61) and directly reversing the EMT phenotype, which in turn suppresses thyroid cancer migration. Conversely, REX1 significantly enhances EMT-dependent migration by activating the JAK2/STAT3 signaling pathway and may indirectly promote the formation of a pro-angiogenic microenvironment (62), further confirming the synergistic role of EMT and angiogenesis in thyroid cancer.

Follicular thyroid cancer (FTC) is associated with a poor prognosis due to its high tumor proliferative activity. In FTC, vimentin expression is low. Single-cell RNA sequencing of FTC samples revealed that UBE2C is significantly upregulated, paradoxically enhancing cell proliferation. UBE2C mediates K29-linked ubiquitination, leading to vimentin degradation, but inhibits cell migration and invasion through EMT regulation.

### 3.3 EMT and colonization of TC

Colonization refers to the process by which circulating tumor cells (CTCs) reside and proliferate in a distal organ. Specifically, after dislodging from the primary tumor and entering the circulation, tumor cells adhere to the vascular endothelium of the distal organ and subsequently extravasate into the parenchymal tissues for dissemination (11). Through the reversal of epithelial-mesenchymal transition, known as mesenchymal-epithelial transition (MET), tumor cells ultimately colonize the distal organ and develop into metastatic foci. CTCs extravasate and migrate into the organ parenchyma through cellular and molecular mechanisms, a process that may involve EMT. For example, the expression of Twist1 or Snail1 promotes tumor cell extravasation, suggesting that EMT may facilitate both extravasation and initial colonization (63). PRRX1, a paired homoeobox factor 1 transcription factor, acts as a regulator of EMT and modulates migration and invasive properties during EMT. During the reversal from EMT to MET, the expression of Twist and PRRX1 decreases, while the expression of other biomarkers (e.g., EGFR and cMET) increases, thereby regulating cancer cell colonization (4) (**Figure 1**).

In the cascade of thyroid cancer metastasis, the distal colonization capacity of tumor cells is closely associated with the dynamic regulation of EMT. Notably, despite follicular thyroid cancer exhibiting high proliferative activity and poor prognostic features, its metastatic colonization ability is subject to specific regulation. Single-cell RNA sequencing has revealed that aberrant upregulation of UBE2C in FTC leads to vimentin degradation via K29-linked ubiquitination. This unique molecular mechanism enhances tumor cell proliferation while impairing cell migration and invasion by inhibiting the EMT process (64). This finding indicates that metastatic colonization in thyroid cancer depends not only on EMT activation but also on a delicate balance between EMT and proliferative activity: while strong EMT inhibition can limit initial invasion, it may paradoxically promote the final colonization of metastatic foci by maintaining the epithelial phenotype. This bidirectional role of EMT regulation offers novel insights into understanding the organ-specific colonization patterns of thyroid cancer metastasis.

In the cascade of tumor metastasis, ncRNAs are closely associated with the EMT in thyroid cancer. MicroRNAs and long non-coding RNAs have been identified as key regulators of the EMT process, and some of these molecules are also implicated in the proliferation of cancer stem cells (CSCs) (65). For instance, the downregulation of LOC389641, a long non-coding RNA (lncRNA) located in the p21 region of chromosome 8, promotes proliferation, wound healing, clonal formation, migration, and invasion of papillary thyroid carcinoma (PTC) cells by modulating the EMT process (66).

## 4 Non-coding RNAs

Non-coding RNAs are RNA molecules that do not code for proteins. They perform diverse functions in cells and play crucial roles in various biological processes, including gene expression regulation, cell signaling, and disease development. Based on their size and function, ncRNAs can be classified into several categories, such as long non-coding RNAs (lncRNAs), microRNAs (miRNAs), and circular RNAs (circRNAs).

### 4.1 MiRNAs and TC

**Figure 2:**
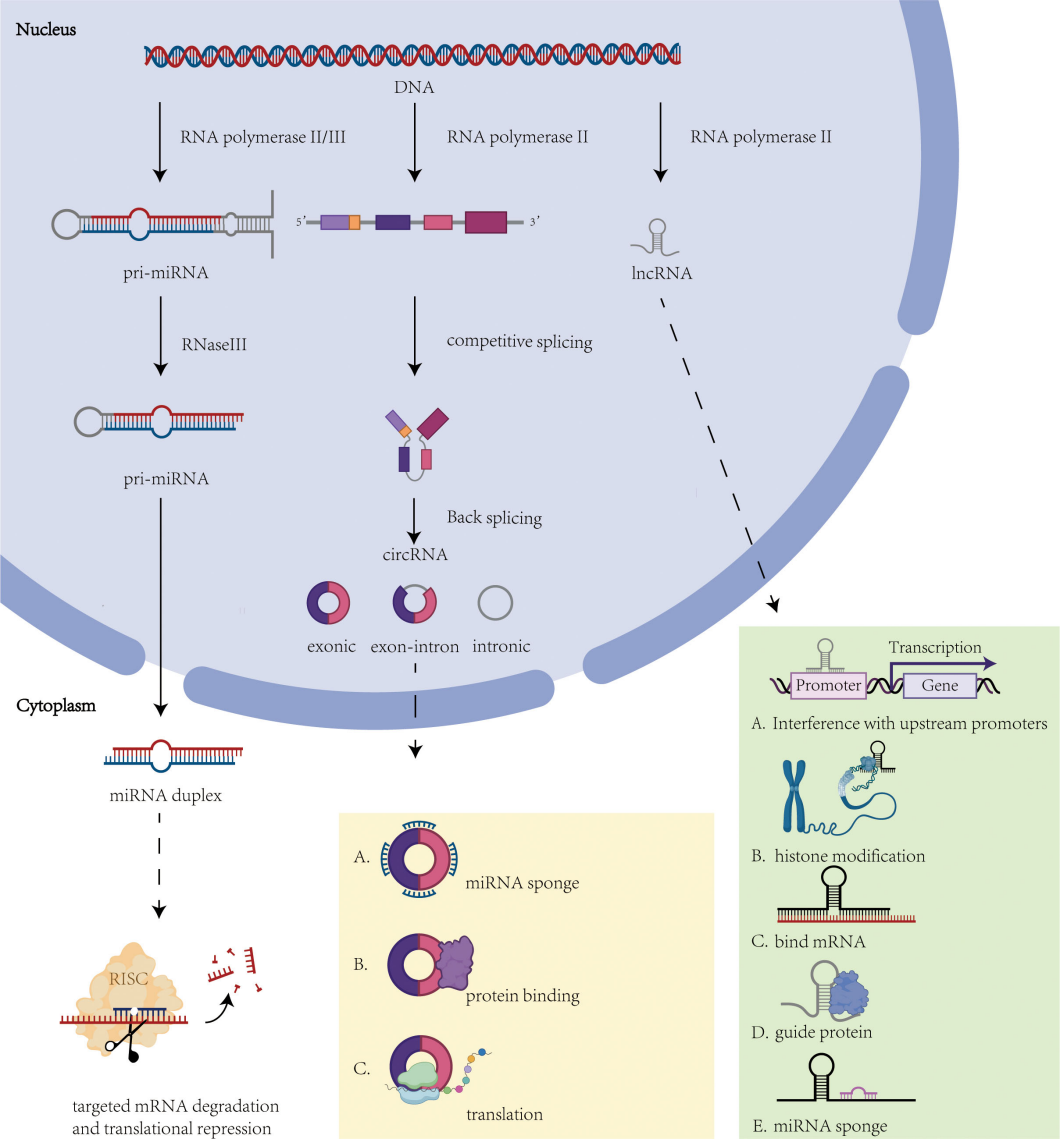
Overview of ncRNA biogenesis and function. This figure illustrates the molecular mechanisms underlying the biogenesis and function of non-coding RNAs. Solid arrows indicate the biogenesis pathways of ncRNAs, while dashed arrows indicate the roles played by ncRNAs. (1) miRNA mechanism of action: regulation of gene expression through miRISC. (2) CircRNA mechanism of action: act as miRNA sponges, regulate protein interactions or signaling pathways. (3) lncRNA mechanism: interferes with upstream promoter transcription, histone modification, interferes with mRNA, binds proteins and acts as miRNA sponge.

MicroRNAs (miRNAs) are a class of highly conserved endogenous small noncoding RNA molecules, typically comprising approximately 22 nucleotides (67, 68). miRNAs are transcribed from DNA by RNA polymerase II or III to generate primary miRNAs (pri-miRNAs) (69). In the nucleus, pri-miRNAs are processed by a complex containing RNase III enzyme, forming precursor miRNAs (pre-miRNAs) with a stem-loop hairpin structure (approximately 60–70 nucleotides). Subsequently, pre-miRNAs are transported to the cytoplasm and further processed by another complex to form mature miRNA duplexes (70). Mature miRNAs bind to target mRNAs through the miRNA-induced silencing complex (miRISC), inhibiting translation or promoting mRNA degradation, thereby regulating gene expression. In malignant tumors, miRNA expression dysregulation plays a critical role in cancer development. For instance, overexpression of miR-221 and miR-222 in gastrointestinal, hepatocellular, and thyroid cancers can target and suppress tumor suppressor genes. Additionally, miRNAs are widely involved in regulating various biological processes, including the cell cycle, cell proliferation, differentiation, migration, apoptosis, and immune response (71) (**Figure 2**).

Increasing evidence indicates that miRNAs serve as key biomarkers in the occurrence, progression, detection, and prognosis of TC. miR-493-5p targets METTL3, whose expression level correlates positively with the degree of thyroid cancer differentiation and whose low expression is closely associated with tumor progression and poor prognosis. METTL3 regulates PAX8 expression via m6A modification, thereby affecting the differentiation and chemosensitivity of thyroid cancer cells (72). miR-31-5p is significantly upregulated in papillary thyroid cancer tissues and in K1 cells harboring the BRAF p.V600E mutation. This miRNA promotes cell proliferation by inducing the expression of EMT markers and upregulating the YAP/β-catenin signaling axis, and is closely associated with cell adhesion, migration, and invasion (73). miRNAs regulate key signaling pathways and target genes (e.g., SNAI1, PSMD10) to exert dual roles (cancer-promoting or cancer-suppressing) in thyroid cancer development (74). Abnormal changes in their expression profiles provide potential markers for the early diagnosis, prognostic assessment, and targeted therapy of thyroid cancer. For example, developing small molecule inhibitors targeting SNAI1 or KIAA1199 based on the regulatory networks of miR-199a-5p, miR-486-5p, and miR-654-3p may represent a novel therapeutic strategy (75–77). These findings offer new targets for thyroid cancer treatment.

### 4.2 CircRNA and TC

Circular RNAs (circRNAs) are covalently closed single-stranded RNA molecules transcribed by RNA polymerase II (Pol II). These non-coding RNAs originate from precursor mRNAs that undergo exon/intron sequence rearrangement via spliceosome-mediated reverse splicing, forming characteristic closed-loop structures (78, 79). CircRNAs exhibit pleiotropic regulatory functions within tumor microenvironments, including modulation of cellular invasiveness, angiogenesis, EMT, and chemoresistance (80, 81) (**Figure 2**).

The molecular mechanisms of circRNAs in thyroid carcinogenesis are classified into four principal categories: miRNA Sponge Effect:CircRNAs competitively sequester miRNAs through complementary base pairing, thereby attenuating miRNA-mediated post-transcriptional repression of target mRNAs and upregulating oncogene expression. Key examples include: circPVT1 aberrantly overexpressed in medullary thyroid carcinoma, circPVT1 promotes tumor proliferation and metastasis by sponging miR-455-5p to activate the CXCL12 signaling axis (82).circ_0003747 demonstrates elevated expression in thyroid carcinoma cells, functioning as a molecular sponge for miR-338-3p. Mechanistically, miR-338-3p suppresses tumor progression by directly targeting the 3’UTR of PLCD3 – an oncogene validated in thyroid cancer cell lines (83). circFAT1(e2) specifically upregulated in papillary thyroid carcinoma (PTC), circFAT1(e2) enhances ZEB1 expression via miR-873 sequestration, driving PTC cell proliferation, migration, and invasion (84); Protein Trafficking Regulation:CircRNAs facilitate proper protein folding and structural stabilization through spatial conformational modifications while competitively inhibiting physiological protein-ligand interactions.; RNA-Protein Complex Assembly:CircRNAs dynamically interact with specific proteins to form functional ribonucleoprotein complexes, critically modulating signaling networks during thyroid oncogenesis.;Translational Control: Certain circRNAs recruit translational machinery to regulate protein synthesis. For instance, hsa_circ_0006943 binds CSNK2A1, enhancing its interaction with Akt to activate the PI3K-Akt pathway and induce EMT, thereby accelerating thyroid cancer progression (85).

Collectively, circRNAs orchestrate multidimensional regulatory circuits governing thyroid cancer initiation, progression, and microenvironmental adaptation. Their mechanistic diversity highlights substantial potential as diagnostic biomarkers and therapeutic targets.

### 4.3 LncRNAs and TC

Long noncoding RNAs (lncRNAs) are a class of noncoding RNAs exceeding 200 nucleotides in length (86). The majority of lncRNAs are transcribed by RNA polymerase II from intergenic or exonic regions (71). These transcripts typically undergo splicing and are modified with a 7-methylguanosine cap at the 5’ end and polyadenylation at the 3’ end (87). lncRNAs exert a pivotal role in thyroid cancer development, progression, and microenvironmental remodeling by modulating gene expression through multiple mechanisms, including epigenetic modification, signaling pathway regulation, and molecular sponge action (**Figure 2**).

Long non-coding RNAs orchestrate core cellular processes including differentiation, autophagy, cell cycle regulation, proliferation-apoptosis balance, invasion-migration capacity, and stemness maintenance through epigenetic networks. In thyroid carcinogenesis, lncRNAs drive malignancy via five principal mechanisms: Cis-Regulatory Modulation: LncRNAs transcribed from upstream promoter regions of protein-coding genes spatially impede downstream transcriptional machinery. Notable examples: LINC00891 activates the EZH2/SMAD2/3 axis to induce epithelial-mesenchymal transition in thyroid carcinoma. Mechanistically, EZH2 overexpression rescues EMT suppression caused by LINC00891 knockdown (88). LINC02454 demonstrates oncogenic activity in thyroid carcinoma through CREB1 phosphorylation-mediated HMGA2 transcriptional activation (89); Chromatin Reprogramming: LncRNAs mediate chromatin topology reorganization and histone modification. Bisulfite sequencing revealed hypermethylation at CpG islands within the MEG3 differentially methylated region (DMR), correlating with its tumor-suppressive silencing in thyroid malignancies (90); RNA Interference Cascade: LncRNA-mRNA duplex formation disrupts canonical splicing. Aberrant splice variants generate endogenous siRNAs via Dicer processing, triggering RNA interference-mediated mRNA degradation. ZNF674-AS1 functions as a ceRNA to suppress thyroid cancer progression by sequestering miR-181a and restoring SOCS4 expression (91); Protein Interaction Networks: LncRNAs scaffold functional ribonucleoprotein complexes. LINC00887 drives papillary thyroid carcinoma (PTC) progression by inducing G1/S phase arrest to promote proliferative advantage and apoptotic resistance; miRNA Sponge Activity: LncRNAs competitively bind miRNAs to derepress oncogenic targets. lncRNA n384546/TUG1 elevated in PTC, these lncRNAs adsorb miR-145-5p to upregulate AKT3 and ZEB1, synergistically enhancing tumor invasiveness (92, 93).

Collectively, lncRNA networks constitute promising diagnostic biomarkers and therapeutic targets in thyroid cancer management.

## 5 Role of EMT-related ncRNAs in TC

### 5.1 EMT-related miRNAs and their association with TC

Epithelial-mesenchymal transition is a critical process in TC invasion and metastasis. miRNAs exert significant regulatory effects by targeting EMT-associated transcription factors or signaling pathway molecules. miR-204-5p is downregulated in TC and directly targets the 3’-UTR of high-mobility group protein 2 (HMGA2), thereby inhibiting its expression and suppressing the MAPK signaling pathway. This action reduces the invasiveness of TPC-1 and BCPAP cells (94). The silencing of HMGA2 significantly inhibits the EMT process, suggesting that miR-204-5p may serve as a potential therapeutic target for TC. Additionally, miR-200b/c targets Rap1b to inhibit the NF-κB/Twist1 signaling pathway, thereby suppressing the proliferation, invasion, migration, and EMT of papillary thyroid carcinoma (PTC) cells (95). Overexpression of Rap1b is closely associated with the malignant progression of TC The BRAFV600E mutation upregulates miR-222-3p, which induces EMT by targeting Snail and is significantly associated with lymph node metastasis in PTC patients (96). miR-199a-5p is significantly downregulated in thyroid undifferentiated carcinoma (ATC), and its overexpression inhibits EMT by targeting Snail, thereby suppressing the migration and invasion of ATC cells both *in vitro* and *in vivo* (97).

**Figure 3:**
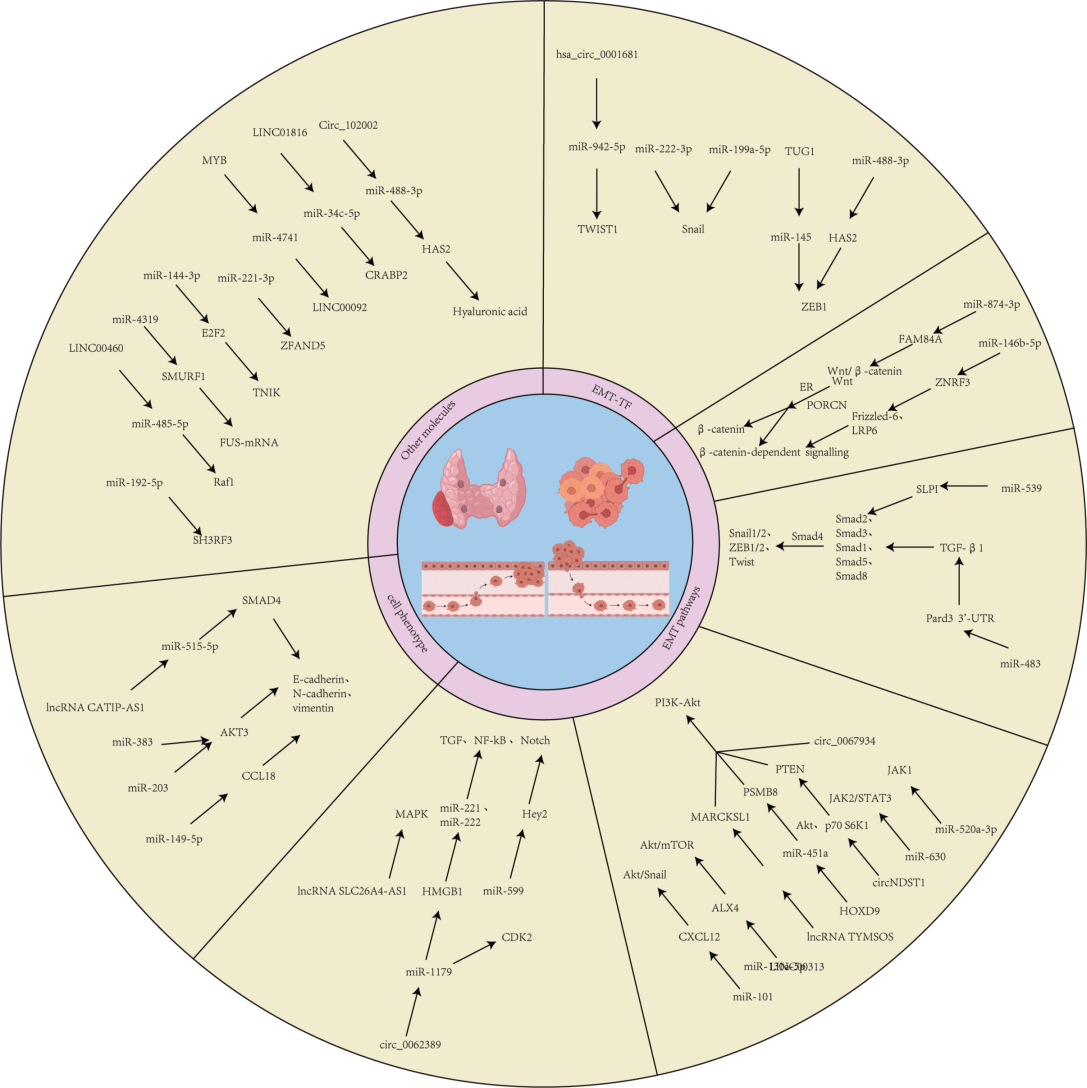
Common regular pathways of ncRNAs. This figure depicts the cross-cutting regular pathways of miRNAs, circRNAs and lncRNAs in EMT transcription factors, EMT signaling pathways, cellular phenotypes and epigenetics.

miRNAs can also affect EMT by modulating signaling pathways. For example, miR-146b-5p enhances Wnt/β-catenin signaling through downregulation of ZNRF3, inducing an EMT phenotype that correlates with extra-thyroidal infiltration and advanced staging in TC (98). In PTC, miR-874-3p is downregulated, and overexpression of its target gene FAM84A activates the Wnt/β-catenin pathway, driving the EMT phenotype (99). miR-483 regulates TGF-β1/Smads signaling by targeting Pard3 (100), and miR-539 activates the TGF-β1/Smads pathway by targeting SLPI (101), thereby regulating EMT in TC. miRNAs also regulate EMT-related markers (e.g., vimentin, E-cadherin) through pathways such as JAK2/STAT3, PI3K/AKT, and Notch, including miR-520a-3p, miR-630, miR-203, miR-101, miR-1271, miR-599, miR-221/222, miR-149-5p, miR-21, etc. (102–108). Additionally, miR-15a, miR-192-5p, miR-4319etc., inhibit EMT by targeting elements such as the Hippo/JNK pathway, SH3RF3, SMURF1, etc., while miR-221-3p and miR-34c-5ppromote EMT and TC metastasis by targeting ZFAND5 and CRABP2 (109–113) (**Figure 3**).

In thyroid cancer, miR-146b-5p significantly affects tumor progression by regulating the EMT process. Studies have shown that miR-146b-5p is overexpressed in papillary thyroid carcinoma (PTC) with lymph node metastasis and downregulates the protein expression of zinc RING finger protein 3 (ZNRF3) by directly targeting its 3′-UTR region (98). Bioinformatics analysis and luciferase reporter assays confirmed that miR-146b-5p inhibits ZNRF3 protein expression by directly binding to its 3′-UTR region. The inhibition of ZNRF3 activates the Wnt/β-catenin signaling pathway, as evidenced by the upregulation of Frizzled-6 and LRP6 receptor expression, which in turn promotes PTC cell migration, invasion, and EMT characteristics (downregulation of the epithelial marker E-cadherin and upregulation of the mesenchymal markers N-cadherin and vimentin). Similarly, TGF-β1 treatment results in altered PTC cell morphology, downregulation of E-cadherin, and upregulation of mesenchymal markers such as Slug, Snail, and Twist. Notably, overexpression of ZNRF3 or the use of Wnt/β-catenin signaling pathway inhibitors can reverse the pro-oncogenic effects of miR-146b-5p, suggesting its potential as a therapeutic target. Although TGF-β1 transiently upregulates miR-146b-5p, its long-term inhibition enhances the proliferation and invasiveness of PTC cells, indicating that miR-146b-5p may balance tumor progression through dynamic regulation (114).

These studies suggest that EMT-related miRNAs influence the aggressiveness of thyroid cancer through a complex regulatory network, providing an important basis for the development of targeted therapies and prognostic markers. Future studies are needed to further validate their clinical potential and explore combination therapy strategies.

### 5.2 EMT-related lncRNAs and their association with TC

During TC progression, lncRNAs)play a key role by regulating EMT-related signaling pathways and target gene expression. lncRNAs influence the EMT process by competitively binding miRNAs or directly regulating signaling pathway molecules. For example, lncRNA TUG1 is significantly upregulated in TC tissues by competitively binding to miR-145 and deregulating its inhibition of ZEB1, thereby promoting ZEB1-mediated EMT process and enhancing the proliferation and migration of thyroid cancer cells (92). DOCK9-AS2 is upregulated in papillary thyroid carcinoma (PTC) by sponge adsorption of miR-1972 CTNNB1 (β-catenin), activating the Wnt/β-catenin signaling pathway, which in turn induces an EMT phenotype and promotes invasion and metastasis of PTC cells (115). TYMSOS, as an oncogenic lncRNA, up-regulates the expression of MARCKSL1 and activates the PI3K/Akt pathway by binding to miR-130a-5p and accelerating the proliferation and EMT process of TC cells (116). LINC00313 inhibits its expression by binding to the ALX4 promoter, activates the AKT/mTOR signaling axis, and promotes TC proliferation, migration, and EMT (117). In addition, oncogenic lncRNA UCA1 targets miR-15a through Hippo and JNK signaling pathways and accelerates the EMT process of TC (113). LINC00460 upregulates Raf1 through sponge adsorption of miR-485-5p, which significantly enhances the EMT phenotype of PTC (118). LINCRNAs can also affect EMT by regulating the expression of oncogenes, such as HOXA-AS2 upregulates S100A4 and ZFAS1 deregulates its repression of downstream oncogenes. HOXA-AS2 enhances cancer cell proliferation, migration, and EMT through sponge adsorption of miR-520c-3p (119). LncRNA ZFAS1 enhances cancer cell proliferation, migration, and EMT by targeting miR-302a-3p (120). LINC01816 is highly expressed in TC tissues and may promote CRABP2 overexpression through sponge adsorption of miR-34c-5p, inducing EMT and cancer cell metastasis (112) (**Figure 3**).

Some long non-coding RNAs function to inhibit EMT. For example, SLC26A4-AS1 is lowly expressed in papillary thyroid carcinoma (PTC), and its overexpression inhibits the expression of EMT-related genes by activating TP53 and inactivating the MAPK pathway, thereby reducing cell migration and invasion (121). Additionally, CATIP-AS1 inhibits TC metastasis by reversing the expression of EMT markers (e.g., E-cadherin and N-cadherin) through the upregulation of SMAD4 by inhibiting miR-515-5p (122). These studies suggest that lncRNAs have dual regulatory roles in thyroid cancer, acting as both tumor suppressors and oncogenic factors, and their clinical translational potential needs to be further verified.

LncRNA RMST expression is significantly reduced in ATC and only slightly reduced in DTC. RMST may inhibit the expression of EMT markers (e.g., SLUG, TWIST1, NES1) by regulating the Wnt/β-catenin, Notch, and Hedgehog signaling pathways and significantly reduce the expression of stem cell markers (e.g., OCT4, SOX2, NANOG) (123), thereby suppressing the aggressive phenotype of ATC cells. SOX2 is an important transcription factor associated with stem cell properties and tumor aggressiveness. RMST may function by directing translational and post-translational modifications, including DNA methylation and SUMOylation (124) and its expression is negatively correlated with SOX2 and acts by helping Sox2 transcription factors bind to their target promoters (125). LncRNAs play a key role in thyroid cancer development, metastasis, and drug resistance through multidimensional regulation of EMT-related pathways and target genes. Future studies are needed to further validate their clinical potential and develop precise lncRNA-based therapeutic strategies.

### 5.3 EMT-related circRNAs and their association with TC

In recent years, the regulatory role of circRNAs in the EMT of TC has garnered increasing attention. circRNAs influence the EMT process in TC by acting as molecular sponges for miRNAs, modulating key signaling pathways (e.g., PI3K/AKT, HAS2/ZEB1), or regulating EMT-related transcription factors (e.g., TWIST1, ZEB1). For instance, hsa_circ_0001681 is upregulated in TC and functions as a sponge for miR-942-5p, thereby relieving the inhibitory effect of miR-942-5p on TWIST1 (126). As a core transcription factor of EMT, TWIST1 upregulation suppresses E-cadherin expression and induces mesenchymal marker expression, ultimately promoting the EMT process and tumor progression in TC (25). Circ_102002 negatively regulates miR-488-3p expression in papillary thyroid carcinoma (PTC) by acting as a sponge. The target gene of miR-488-3p, HAS2, accelerates the EMT process by synthesizing hyaluronic acid to activate ZEB1. Aberrant expression of circ_102002 enhances HAS2/ZEB1 axis activity, thereby promoting the migration and invasion of PTC cells (127). Additionally, circ_0067934 is significantly overexpressed in thyroid tumors and induces an EMT phenotype by activating the PI3K/AKT signaling pathway. This action promotes cancer cell proliferation, migration, and invasion while inhibiting apoptosis. Its effects are closely related to EMT markers, such as upregulation of N-cadherin and downregulation of E-cadherin (128). In contrast, circGLIS3 expression is reduced in TC and upregulates AIF1L expression by binding to miR-146b-3p, thereby reducing its inhibition of AIF1L mRNA. AIF1L may affect cellular phenotypic transition and inhibit the malignant progression of TC by regulating EMT-related genes (129). Some circRNAs are associated with treatment resistance. For example, circ007293, delivered to PTC cells via exosomes, competitively binds miR-653-5p and relieves the inhibition of PAX6 by miR-653-5p. Activation of PAX6 promotes the EMT process and enhances cell invasion and migration. Silencing circ007293 significantly inhibits its pro-oncogenic effects (130). circPTPRM acts as a sponge for miR-885-5p and significantly enhances the malignant phenotype of TC (131) (**Figure 3**). These studies suggest that targeting circRNAs may be a novel strategy to reverse TC resistance (**Tables 1**, **2**).

**Table 1:** The functions of EMT-related ncRNAs in TC.

ncRNA	Target or the Whole Pathway	Functions in TC	Reference
miR-204-5p	HMGA2	Invasion (-) EMT (-)	(94)
hsa_circ_0001681	TWIST1	EMT (-)	(126)
miR-200b/c	Rap1b	Proliferation (+) Invasion (+) Metastasis (+) Apoptosis (-) EMT (+)	(95)
miR-222-3p	Snail	Metastasis (+) Lymph node metastasis (+) EMT (+)	(96)
miR-199a-5p	Snail1	Invasion (-) Metastasis (-) EMT (-)	(97)
miR-145	ZEB1	Proliferation (+) Metastasis (+) EMT (+)	(92)
miR-488-3p	HAS2	EMT (+)	(127)
miR-146b-5p	Wnt/β-catenin	Invasion (+) EMT (+)	(98)
miR-874-3p	FAM84A	EMT (+)	(99)
lncRNA DOCK9-AS2	miR-1972	Proliferation (+) Invasion (+) Metastasis (+) EMT (+)	(115)
miR-483	Pard3	Invasion (+) Metastasis (+) EMT (+)	(100)
miR-539	TGF-β1/Smads	Proliferation (-) Apoptosis (+) EMT (-)	(101)
miR-520a-3p	JAK/STAT	Invasion (-) Metastasis (-) EMT (-)	(102)
miR-630	JAK2/STAT3	Invasion (-) Metastasis (-) EMT (-)	(103)
miR-451a	PSMB8	Proliferation (+) Invasion (+) Metastasis (+) EMT (+)	(148)
circ_0067934	PI3K/AKT	Proliferation (+) Invasion (+) Metastasis (+) Apoptosis (-)	(128)
miR-21	PI3K/AKT	EMT (+)	(149)
lncRNA TYMSOS	PI3K/Akt	EMT (+)	(116)
miR-26a	PI3K/AKT	Proliferation (-) Invasion (-) Metastasis (-) EMT (-)	(150)
miR-203	AKT3	Invasion (+) Metastasis (+) EMT (+)	(104)
LINC00313	AKT/mTOR	Proliferation (+) Invasion (+) Metastasis (+) EMT (+)	(117)
miR-101	CXCL12	Proliferation (-) Invasion (-) Metastasis (-) Apoptosis (+) EMT (-)	(105)
miR-1271	IRS1	Proliferation (-) Invasion (-) Metastasis (-) EMT (-)	(106)
miR-599	Notch	Proliferation (+) Invasion (+) Metastasis (+) Apoptosis (-) EMT (+)	(107)
lncRNA UCA1	miR-15a	Proliferation (+) EMT (+)	(113)
circ_0062389	miR-1179	Proliferation (+) Apoptosis (-) EMT (+)	(151)
lncRNA SLC26A4-AS1	MAPK	Apoptosis (+) EMT (-)	(121)
miR-31	SOX11、ERK、Akt	Proliferation (-) Invasion (-) Metastasis (-) EMT (-)	(152)
miR-149-5p	CCL18	Proliferation (-) Metastasis (-) E-cadherin (+) N-cadherin (-) vimentin (-) EMT (-)	(108)
miR-181a	KLF15	Proliferation (-) Metastasis (-) EMT (-)	(58)
miR-203	AKT3	E-cadherin (+) vimentin (-) EMT (-)	(104)
circGLIS3	miR-146b-3p	EMT (+)	(129)
lncRNA CATIP-AS1	miR-515-5p	Proliferation (-) Metastasis (-) EMT (-)	(122)
miR-192-5p	SH3RF3	Invasion (-) Metastasis (-) EMT (-)	(110)
lncRNA HOXA-AS2	miR-520c-3p	Invasion (+) Metastasis (+) EMT (+)	(119)
circPTPRM	miR-885-5p	EMT (+)	(131)
LINC00460	Raf1	Proliferation (+) Metastasis (+) EMT (+)	(118)
miR-4319	SMURF1	Proliferation (-) Metastasis (-) EMT (-)	(111)
miR-144-3p	E2F2	EMT (-)	(138)
circ007293	miR-653-5p	Proliferation (+) Invasion (+) Metastasis (+) EMT (+)	(130)
lncRNA ZFAS1	miR-302a-3p	Proliferation (+) Invasion (+) Metastasis (+) EMT (+)	(120)
miR-221-3p	ZFAND5	Proliferation (+) Invasion (+) Metastasis (+) EMT (+)	(109)
LINC01816	CRABP2	Invasion (+) Metastasis (+) EMT (+)	(112)
INC00313	miR-422a	EMT (+)	(153)

+ means promoting, - means inhibiting.

**Table 2:** Key EMT-associated ncRNAs in thyroid cancer.

ncRNA	Target or regulated molecule	Signaling Pathway	Functional Role in TC	Reference
miR-146b-5p	ZNRF3	Wnt/β-catenin	Promotes invasion and EMT by enhancing Wnt signaling	(154)
miR-200b/c	EGFR	Rho/ROCK signaling	Activates EGFR, enhances proliferation, invasion, and EMT.	(155)
miR-539	SLPI	TGF-β1/Smads	Inhibits EMT and cell proliferation via TGF-β1/Smads suppression.	(101)
miR-630	JAK2/STAT3	JAK2/STAT3	Suppresses EMT and metastasis by inhibiting JAK2/STAT3 pathway.	(103)
miR-204-5p	HMGA2	MAPK	Reduces invasion and EMT by targeting HMGA2 and inhibiting MAPK signaling.	(94)
miR-21	PI3K/AKT	PI3K/AKT	Promotes EMT and cell proliferation via PI3K/AKT activation.	(156)
miR-874-3p	FAM84A	Wnt/β-catenin	Downregulated in PTC; FAM84A overexpression activates Wnt/β-catenin and EMT.	(99)
circPVT1	miR-195	Wnt/β-catenin	Drives PTC growth and metastasis via Wnt/β-catenin signaling.	(157)
DOCK9-AS2	miR-1972/CTNNB1	Wnt/β-catenin	Activates Wnt pathway by upregulating CTNNB1, promoting PTC invasion.	(115)
LINC00313	miR-422a, miR-4429	AKT/mTOR	Activates AKT/mTOR signaling to drive EMT and metastasis.	(153, 158)
CATIP-AS1	miR-515-5p/SMAD4	TGF-β/Smads	Inhibits EMT by upregulating SMAD4 and suppressing TGF-β signaling.	(122)

## 6 Potential clinical applications of ncRNA in thyroid cancer

Thyroid surgery, radioactive iodine therapy, and TSH suppression remain the mainstay treatments for thyroid cancer (42). Preliminary studies suggest that total thyroidectomy may be more cost-effective and efficacious than hemithyroidectomy (93, 94). For patients with high-risk thyroid cancer, total thyroidectomy combined with adjuvant radioactive iodine therapy is typically required. In contrast, for low-risk thyroid cancers measuring 1–4 cm in diameter, hemithyroidectomy may be considered (132, 133). Radioactive iodine (RAI) is the first-line treatment for metastatic thyroid cancer. Although surgery and RAI therapy are effective for most patients with DTC, approximately 60% of patients with aggressive metastatic DTC are resistant to RAI therapy and have a poor prognosis (134). Mechanisms of RAI resistance include genetic mutations (e.g., BRAF V600E mutation, TERT promoter mutation), dysfunction of iodine-transporting proteins (e.g., NIS), and disturbances in the tumor microenvironment (TME).

Strategies to overcome RAI resistance include (1): tyrosine kinase inhibitors (TKIs) that improve RAI uptake by inhibiting multiple receptors (e.g., VEGFR, c-KIT, PDGFRα/β; (2) drugs that restore RAI affinity (e.g., BRAF inhibitors, HDAC inhibitors, MEK inhibitors (135); (3) drugs that induce redifferentiation of DTC cells and restore NIS expression and iodine uptake capacity (e.g., retinoic acid [RA], all-trans retinoic acid [ATRA]); and (4) the use of nanoparticles as drug carriers to increase RAI concentration in tumor tissues (136). Although TKI treatment improves progression-free survival (PFS) in some patients, the complete remission rate remains low, and patients may discontinue treatment due to adverse effects. Therefore, further research into the molecular mechanisms of RAI resistance and the development of more effective therapeutic strategies, such as drugs targeting specific gene mutations or fusions and improved drug delivery through nanotechnology, are needed.

ncRNAs play a significant role in the clinical management of thyroid cancer patients and can serve as biomarkers for diagnosis, prognosis, and treatment. For example, downregulation of PAR5 lncRNA in undifferentiated ATC serves as a marker of aggressiveness, whereas overexpression of miR-146b in PTC is associated with a poorer prognosis (137). Additionally, ncRNA assessment may help predict therapeutic response and optimize treatment strategies.

Non-coding RNAs play a significant role as potential biomarkers and therapeutic targets in TC therapy. For example, Yi et al. found that miR-144-3p significantly inhibits the proliferation, migration, invasion, and EMT of thyroid cancer cells by downregulating the expression of E2F2 and TNIK, as detected by quantitative reverse transcription-polymerase chain reaction (qRT-PCR) (138). Additionally, overexpression of ciRS-7 promotes the progression of papillary thyroid carcinoma (PTC) by regulating the miR-7/EGFR axis and is closely associated with aggressive clinicopathological features, such as tumor size and lymph node metastasis (139). Orlandella FM et al. found, through real-time quantitative polymerase chain reaction (qPCR) and immunohistochemistry analyses, that restoring JAM-A expression can inhibit the malignant features of thyroid undifferentiated carcinoma (ATC) cell lines, including cell proliferation, motility, and trans-endothelial migration, suggesting that JAM-A could be a potential therapeutic target for thyroid cancer (140). JAM-A expression can be increased and its function restored by modulating gene expression (e.g., via transcription factors or miRNAs) or by activating the PI3K/Akt and MAPK signaling pathways.

Moreover, ZEB1 plays a crucial role at the forefront of tumor invasion, and CD73 is involved in regulating ZEB1 non-coding RNA through its 3’UTR, which in turn affects the progression of PTC. Studies have shown that siRNA molecules targeting CD73 and ZEB1, in combination with RGD-coupled chitosan lactate nanoparticles, can effectively treat thyroid cancer and improve prognosis (141). Metformin shows potential therapeutic value by inhibiting the mTOR signaling pathway and targeting JAK2 in the JAK2/STAT3 signaling pathway, thereby effectively inhibiting the proliferation, migration, and EMT of thyroid cancer cell lines (142, 143). Meanwhile, Tnnt1 significantly promotes the proliferation, colony formation, migration, invasion, and EMT of PTC cells by activating the p38/JNK signaling pathway (144). Silymarin inhibits the proliferation, metastasis, and invasion of PTC cells by modulating the FN1/AKT signaling pathway and inhibiting the EMT process. Additionally, researchers are exploring immunotherapies for thyroid cancer; for example, silymarin reverses the immunosuppressive state of thyroid cancer by regulating the expression levels of immune checkpoint genes (145).

An in-depth investigation of the regulatory mechanisms of ncRNAs in the EMT process of thyroid cancer is expected to lead to breakthroughs in the discovery of early screening biomarkers and precise therapeutic targets, thereby improving the prognosis, prevention, and treatment of thyroid cancer.

## 7 Summary

TC is one of the major malignancies of the endocrine system. In recent years, research has primarily focused on the effects of growth factors, transcription factors, non-coding RNA (ncRNA), DNA methylation, and other regulatory factors on the EMT, as well as the mechanisms underlying EMT in thyroid cancer invasion and metastasis. In cancer therapy, targeting ncRNAs involved in the EMT process has emerged as a promising intervention strategy, potentially preventing or inhibiting the deleterious effects of tumors.

Targeting ncRNAs exerts a crucial regulatory role in cancer progression and may represent a novel therapeutic strategy against thyroid cancer in the future. There are two main approaches to targeting ncRNAs: the first involves inhibiting tumor progression by suppressing oncogene-associated overexpressed ncRNAs, while the second involves inhibiting cancer development by activating or upregulating tumor suppressor gene-associated ncRNAs. For example, downregulation of miR-146b-5p or upregulation of miR-874-3p inhibits the Wnt/β-catenin signaling pathway, thereby blocking EMT activation and inhibiting thyroid cancer progression (99). Additionally, miR-99a-3p is downregulated in PTC tissues and cells, whereas upregulation of miR-99a-3p inhibits EMT, resistance to anoikis, and the migratory and invasive capabilities of PTC cells. ITGA2 has been identified as a downstream effector of GRP94. Overexpression of miR-99a-3p suppresses ITGA2 expression, while overexpression of GRP94 reverses the inhibitory effect of miR-99a-3p on PTC metastasis (146). Different types of ncRNAs can inhibit the EMT process by regulating various transcription factors, signaling pathways, or cellular markers. Therapeutic strategies targeting EMT-associated ncRNAs can be developed by designing specific small molecules or drugs to target these ncRNAs, thereby inhibiting the EMT process and reducing tumor invasion and metastasis (68, 147).
